# Using intervention time series analyses to assess the effects of imperfectly identifiable natural events: a general method and example

**DOI:** 10.1186/1471-2288-6-16

**Published:** 2006-04-03

**Authors:** Stuart Gilmour, Louisa Degenhardt, Wayne Hall, Carolyn Day

**Affiliations:** 1 National Drug and Alcohol Research Centre, University of New South Wales, Sydney, NSW 2052, Australia; 2 School of Population Health, University of Queensland, Herston, Queensland 4006, Australia; 3 National Centre in HIV Epidemiology and Clinical Research, University of New South Wales, Level 2, 376 Victoria Street, Darlinghurst, NSW 2010, Australia

## Abstract

**Background:**

Intervention time series analysis (ITSA) is an important method for analysing the effect of sudden events on time series data. ITSA methods are quasi-experimental in nature and the validity of modelling with these methods depends upon assumptions about the timing of the intervention and the response of the process to it.

**Method:**

This paper describes how to apply ITSA to analyse the impact of unplanned events on time series when the timing of the event is not accurately known, and so the problems of ITSA methods are magnified by uncertainty in the point of onset of the unplanned intervention.

**Results:**

The methods are illustrated using the example of the Australian Heroin Shortage of 2001, which provided an opportunity to study the health and social consequences of an abrupt change in heroin availability in an environment of widespread harm reduction measures.

**Conclusion:**

Application of these methods enables valuable insights about the consequences of unplanned and poorly identified interventions while minimising the risk of spurious results.

## Background

There are occasions when an unexpected event produces major changes in a process that provide an opportunity to investigate its nature and dynamics. These unplanned interventions can provide unique insights into environmental, sociological or physical phenomena that would be otherwise impossible to obtain, such as, for example, in the distribution of radioactivity after the Chernobyl disaster and its effect on the human food chain and economic processes [1,2]. Such natural experiments provide important research opportunities for public health, enabling investigations of phenomena which would not otherwise be practical or possible [3]. In such circumstances the method often involves the analysis of the impact of the event on the best available regularly collected surveillance data on outcomes of interest using intervention time series analysis (ITSA).

### Intervention time series analysis

ITSA has become a standard statistical method for assessing the impact of an intervention (usually a planned policy change) on a time series of relevant outcome indicators. In this paper we describe how ITSA can be adapted to model the impact of an unexpected event whose point of onset cannot be accurately identified. We illustrate the method in the context in which it was developed – modeling the health and social consequences of an abrupt decline in heroin availability that occurred in Australia in 2001–2002. This event provided a unique opportunity to investigate the impact of supply reduction on indicators of an illegal, largely hidden and poorly understood behaviour: illicit heroin injecting [4].

The aim of this paper is to describe how these methods can be applied to *indicator data *to construct and test plausible theories of the consequences of a poorly identified unplanned intervention in ways that minimize the risk of drawing erroneous conclusions. The aim of this paper is to describe how these methods can be applied to indicator data to construct and test plausible theories of the consequences of a poorly identified unplanned intervention in ways that minimize the risk of drawing erroneous conclusions. We identify the major challenges that arise in applying standard ITSA methods to the analysis of such natural experiments and suggest methods for overcoming them. The Australian heroin shortage, a unique and unexpected event, is used for illustrative purposes only, detailed results of the analyses can be found elsewhere [4-8]. The emphasis of this paper is on the logic of the approach rather than the details of the statistical methods which readers can readily find elsewhere [9].

### The Australian heroin shortage

In early 2001, heroin users, local police and those working in drug treatment services reported a sudden decline in the availability of heroin in Sydney and Melbourne. These reports were quickly investigated by conducting timely surveys of heroin users [10-12]. The 2001 Illicit Drug Reporting System (IDRS) – Australia's strategic early warning system for monitoring trends in illicit drug markets – subsequently revealed that there had been an abrupt reduction in the availability and purity of heroin and an increase in heroin price in all major Australian heroin markets [11,12]. The reduction in availability was most marked from January to April 2001 [13] with the most frequently suggested "onset" of the shortage being January 2001. Although availability appears to have returned for regular injecting drug users (IDU), the price remains higher and street level purity remains significantly lower than the levels of the 1990s [14,15]. We refer to this phenomenon as the "heroin shortage".

This abrupt and sustained reduction in heroin supply, coupled with sophisticated monitoring of key parameters of the drug market, drug use, and its harms, provided a unique situation in which to carefully examine the impact of a large change in supply upon drug use and drug-related harms. This was carried out through a detailed 18 month study of the causes, course and consequences of the heroin shortage [4]. Such a natural experiment provided an opportunity to inform Australian policy-makers about the consequences of substantially reducing heroin use, a policy goal that has only rarely been achieved.

## Method: Modeling the effects of planned and unplanned interventions

Intervention time series analysis models the effect of known interventions on data of counts of events collected regularly in time [16,17]. Such data often show *serial dependence*, in which observations are correlated with previous observations separated by some time delay (typically 1 month, or 12 months in the case of seasonal effects). Well-established methods for handling such data enable the event to be modeled as a simple function (the *intervention*) with which the data series is correlated. The effect of a planned event can be estimated after adjusting for serial dependence and seasonal effects, by using linear models to quantify increases or decreases in the mean level or slope of the series. Typically such analyses use Auto-regressive Integrated Moving Average (ARIMA) models to handle the serial dependence of the residuals of a linear model, which is estimated either as part of the ARIMA process or through a standard linear regression modeling process [9,17]. All such time series methods enable the effect of the event to be separated from general trends and serial dependencies in time, thereby enabling valid statistical inferences to be made about whether an intervention has had an effect on a time series.

Standard ITSA requires that the point of occurrence of the event is known and a specific functional form be specified for the effect that the event has on the time series. This functional form represents the researchers' assumptions about the likely effects of the event, and the serial dependence between the function and the data series (in the form of auto-correlation functions) is used to estimate by how much the effect lagged the event. In the case of models with complex linear terms, or where the data series shows different behaviour (such as upward and downward trends) in different periods of time, standard linear models can be fit with the intervention included as a variety of different functions. Where the data shows stable behaviour over the entire period of the study (outside of the period due to the intervention) ARIMA models can be fit directly to the data series, or to a series which has been suitably treated to induce stationarity. In this case the intervention can be modeled as a simple function such as a step or pulse and the precise short term behaviour of the time series after the event can be estimated from the data by applying transfer functions to this simple function. These transfer functions *modify *simple step, pulse or change of slope functions to represent a range of possible effects that include a long-term change which occurs after a period of 'ramping up', and a sudden increase followed by (possibly oscillating) 'exponential decay'. Such a modeling process separates the primary question – 'was the change temporary or permanent' – from the secondary question: 'what was the precise shape of the identified short or long term change?' The test of the step or pulse term is used to answer the first question and the transfer functions are used to answer the second question. The added versatility of these transfer functions makes ARIMA modeling of this sort preferable to linear models with separate estimation of the serial dependence of the residuals, which provide less flexibility for modeling the intervention. Nonetheless, both methods provide a suitable and statistically valid method for estimating the effect of the intervention and (in the case of ARIMA models) the details of the short term behaviour of the series after the intervention.

All such ITSA methods require a model-building process in which key features of the model cannot be specified ahead of the data – the functional form is often defined by the researchers after inspection of the data, and a variety of candidate transfer functions may be fitted to the data. Model-building techniques have been developed for choosing between candidate functions at both stages using considerations such as model parsimony, minimization of information criteria, or ANOVA-type tests that are penalized for model complexity [9].

In situations where the time of onset of the event is not clear and the nature of its effect is poorly understood, the ITSA model-building process is more exploratory than confirmatory. Even planned experiments with ITSA methods can face difficulties of interpretation and model-building – in some cases, for example, behaviours change in anticipation of the event, other events can occur simultaneously with the intervention, or the effect may be unexpected or delayed and a wide range of candidate functions may be available to describe its effects. These challenges are complicated further when the experiment is unplanned and the point of occurrence of the event is not clearly defined. Many researchers recommend against the use of ITSA in such circumstances [18]. We have been unable to identify any major published studies which perform ITSA on unplanned events with poorly identified time points, but the unique nature and large scale of unplanned events such as the Australian Heroin Shortage demand an adaptation of these methods in order to better understand the effects of such events on various indicators of drug use and drug-related harm.

The major challenges that need to be addressed are: (1) defining and dating the unplanned event in a way that is not contaminated by knowledge of changes in outcome indicators; (2) selecting an appropriate outcome indicator series to model the impact of the event; (3) statistically modeling the impact of the event; and (4) evaluating rival explanations of the changes that followed the event. We do not deal with the statistical issues in time series analysis, such as assessing stationarity, the number of data points that need to be used, the interpretation of auto-correlation and partial auto-correlation functions, and other aspects of the mechanics of time series modeling. These are well described in standard treatments of ITSA to which the reader is referred [9,16,17].

### (1) Defining and dating an unplanned event

Because the model-building method for ITSA is exploratory, the date of onset of the unplanned event can play an important role in the model-building process. Poorly defined or incorrect dates necessarily lead to a wider range of possible models. If the time period over which the event occurred is sufficiently long it may be necessary to model several different starting times. Since the point of onset of the event is unknown, it must be inferred from data sources that are independent of the time series data that will be used for the ITSA. Additional accuracy can be obtained by reference to other research papers and interviews with experts in the field or individuals who may have had opportunity to observe the event first-hand.

No clear single date of onset could be established for the heroin shortage so a variety of data sources were cross-referenced to establish a *likely *onset date. The clearest evidence of the onset of the shortage came from cross-sectional surveillance surveys of IDU, who reported sudden changes in the availability and price of heroin in late 2000 and early 2001 [10-12]. These reports were confirmed by interviews with Key Informants in police, health, treatment and customs agencies. Information from these sources narrowed the time period for the onset of the shortage to a two month time period (December 2000 or January 2001) [13]. The modal estimate of the onset of the shortage was January 2001.

### (2) Choosing indicator data

The choice of data to be used for ITSA is an important consideration even when the experiment is carefully designed and the theory well understood. Results may be biased if the available data do not represent a properly random sample of all available data, or represents only specific sub-populations of events. The effects of poor choice of data may be exaggerated in a natural experiment whose effect is not clearly understood, since it may have differential effects on different sub-populations, leading to erroneous conclusions. The preferred data for ITSA with poorly defined events should be *indicator data*, which is:

• A complete sample of all recorded events of interest or a properly random subsample of these events;

• Data which was not collected solely for the purpose of monitoring the event, since anticipation or understanding of the event may effect the collection of the data; and

• Recorded in the same way across the entire period of data collection.

The data used in ITSA for assessing the effects of the intervention should not be used to identify its timing. Indicator data often lack information on sub-populations and covariates, but notwithstanding their limited usefulness in the analysis of finely detailed hypotheses they remain of crucial importance in the analysis of major changes due to the types of unplanned events described here.

A wide range of indicators of the consequences of heroin and other drug use were used in the heroin shortage study, including ambulance attendances at suspected heroin overdoses, drug related deaths, heroin possession arrests, property crime, and drug treatment episodes. All indicator data used for the study were drawn from databases maintained by agencies external to the researchers, who collected the data for purposes of routine service evaluation and monitoring rather than for research. All data used represented complete counts of all events recorded anywhere in the jurisdiction/nation, and were typically collected according to strict rules (such as the ICD-10 diagnostic codes) that did not change over the period of the study. An important feature of these indicator data is that they were not used to date the onset of the shortage. The contemporaneous key informant reports from which the date of the onset of the shortage was estimated [11,12] could not be influenced by indicator data because such data were not available to key informants because of time lags in their collection, analysis and publication. For example data on overdose deaths and nonfatal overdoses were not made public for a year or more after the onset of the shortage.

### (3) Modelling the effects of the unplanned intervention

The process of developing a functional form of the intervention in this case study (both as the original step or pulse function, and after necessary modification with transfer functions) was necessarily exploratory. Ideally, in order to reduce arbitrary choices for the functional form representing the event and its associated transfer functions:

• The basic functional form should be identified by prior theory;

• Transfer functions which modify this simple function should be drawn from a small set of simple functions, with the intention *only *of modifying the response to the function; and

• The set of possible transfer functions should be restricted by considerations of parsimony

Transfer functions applied to simple basic functions enable the response of the series to the function to be modeled from the data in ways that limit prior assumptions about the model to very broad statements. This enables hypotheses such as 'the level of the series rose briefly' to be modeled, as opposed to hypotheses such as 'the level of the series rose, and declined exponentially to pre-intervention levels'. Unless there are strong reasons to make prior assumptions about the form of the *response *of the series to the intervention, simple transfer functions should be used to model this response. This would rule out, for example, exponential or trigonometric functions, which put restrictions on the series response to the intervention as well as the intervention's broadest form.

We assumed that the most likely responses of the various indicator series to the heroin shortage would include a combination of one or more of the following: a) a brief change followed by a return to pre-shortage levels; b) a long-term change in level; and c) a change in the slope of the time series. These patterns are easily modeled by combinations of step- and pulse-functions, or by a line with unit slope. All trends were assumed to be zero before the postulated point of onset of the heroin shortage. The particular choice of model and transfer function was then estimated from the cross-correlation function between the posited function and the time series, under the assumption that the transfer function served only to delay onset of the functional form, or to modify its shape over a short period of time. This gave a small set of candidate models from which the best were selected by a combination of the following criteria: smallest Akaike Information Criterion (AIC) and prediction variance, number of significant terms and parsimony.

### (4) Evaluating rival explanations

If the ITSA modeling indicates that there has been a change in the time series the plausibility of attributing the change to the unplanned intervention depends upon the implausibility of rival explanations of the change [16]. This involves a modified form of the logic of quasi-experimentation as outlined by Cook and Campbell [19,20] in which plausible alternative explanations are generated and their plausibility tested against the data. If the timing of the intervention is not clearly defined, the possibility that its effects have a rival explanation increases.

The two most plausible rival explanations in the present case were: (i) that a change occurred in the process of measuring the time series at approximately the same time as the intervention, or the measurement was affected by the intervention; and (ii) that there was confounding by other interventions that occurred concurrently with the intervention that may have affected the time series.

Unplanned events can interfere with ITSA even when the experimental event was planned by the researchers. This possibility needs to be investigated and ruled out by reference to:

• Independent data that provides an insight into the behaviour of the time series;

• Existing research in the field and related fields;

• The views of independent experts not involved in the research project who have an understanding of the dynamics of the process being studied; and

• Documentation, service evaluation, diaries and other operational material from organizations in direct contact with the process of interest.

Organizations and individuals involved in collecting data to be analyzed need to be interviewed to ensure that the data collection methods did not change during the study period.

In the case of the heroin shortage project, the most plausible alternative explanations were ruled out by information collected in:

• Detailed interviews with individuals in positions which provided key insights into the behaviour of heroin markets – so-called "key informants" or "key experts" (KI). For the heroin shortage project, the KI included 58 law enforcement personnel and 105 health professionals who were involved with drug users in the areas containing the major drug markets. The KI were either identified by the researchers or recommended by the agency, depending on their position within the service and their contact with drug users. KI were able to comment from a range of perspectives, including policy, program design and implementation and service delivery.

• Security protected police intelligence documents which included extensive details of the operation and behaviour of drug markets;

• Personal communication with data custodians to ensure that other causes of changes in data (such as coding or recording of events) could be ruled out;

• Structured interviews with injecting drug users over the period 1996–2003 collected as part of a pre-existing drug monitoring system (the Australian Illicit Drug Reporting System [14]); and

• Reviews of external research in all relevant fields, including criminal and public health surveillance.

Plausible rival explanations for the heroin shortage included:

• Ambulance strikes, leading to a reduction in heroin overdose records: Strikes and industrial action were ruled out by communication with Ambulance service management and data custodians;

• Major changes in police activity: Extensive interviews with police key informants from operational to strategic levels in all jurisdictions and federally failed to find any evidence of major changes in police activity that would have explained trends in arrests for heroin-related or property offences. There was no evidence in external research or internal police documents to suggest that there had been any major changes in police activity;

• Changes in treatment: Methadone and buprenorphine treatment were made more accessible over the period during which the indicator data were collected as a result of increased government funding in 1999–2000. However, the rate of increase in numbers of treatment places and number of persons treated was too low to account for the abrupt onset and large reduction in heroin overdose deaths and nonfatal overdoses attended by ambulance staff that occurred immediately after both IDU and KI identified the onset of the heroin shortage.

Key informants in health, law enforcement, treatment and ambulance services reported that the shortage began at the same time and no other major events occurred around the same time as the shortage. It is unlikely that minor changes in a single domain (such as drug treatment) would have large enough impact to be noticed by KI working in all other domains at the same time There was also clear agreement between professional key informants and IDU in the timing of the onset of the event. All of these occurrences suggest that the heroin shortage was a real and identifiable event in the drug markets and that its timing was not confounded by any other event or events that would explain the simultaneous, abrupt and substantial changes in manifold indicators of heroin use.

## Results

The data series on cocaine and heroin possession offences in NSW provide an illustrative example of the use of the heroin shortage natural experiment to confirm a long-standing theory of interaction or competition between these two markets. The cocaine possession offences data were modeled using an ARIMA method. Current theories about drug markets suggested that when heroin became less available there would be increased use of other drugs that were more readily available. Cocaine has traditionally been far less available in Australia than heroin but cocaine's availability has increased in recent years [21]. It was important to see if the reduction in heroin availability was accompanied by an increase in the number of individuals charged with possession of cocaine and a decline in the number charged with heroin offences. Because cocaine use is characterized by bingeing and is a self-limiting habit (due to the high cost and physical exhaustion associated with bingeing), it was expected that any switch from heroin to cocaine injecting would be short-lived.

**Figure 1:**
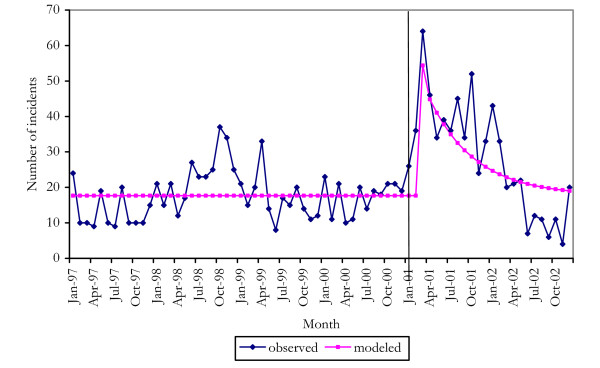
observed and modeled cocaine possession offences.

The effects of the shortage on cocaine were modeled as a pulse function beginning in January 2001. Transfer functions were used to model the slow decay in rates of cocaine possession offences as cocaine users slowly dropped out of the market, and a step function was used to model a possible long-term change in the number of individuals using cocaine either because some individuals found that they preferred this drug, or because excessive use may have led some individuals to cease using it. The final model was a sudden increase two months after onset of the shortage, with a subsequent slow decay to pre-shortage levels (Figure 1). The pulse represented a 207% increase on pre-shortage numbers of possession offences, and the decay term indicated that it took approximately 15 months to return to pre-shortage levels [7].

**Figure 2:**
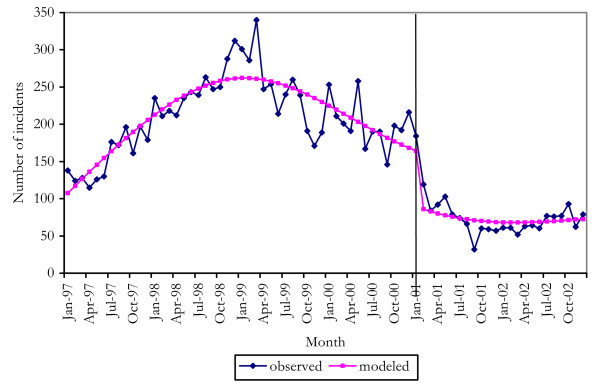
observed and modeled heroin possession offences.

Heroin possession offences were expected to drop sharply from the onset of the shortage, and to remain lower while heroin availability was reduced. The shortage was modeled as a step function. Because heroin possession offences show different behaviour at different time periods, the heroin possession data was modeled using Generalised Least Squares with natural spline smoothers (Figure 2). Several heroin-related series had to be treated in this way, thereby precluding the use of auto-correlation functions to estimate the lag between onset of the shortage and its response in the data series. This additional subjectivity was handled by modeling the functions at only the most common time lags identified in the other time series on which ARIMA analyses could be performed.

The best model was chosen as that which minimized the AIC, had lowest prediction variance and the smallest number viable of knots for the natural spline. Although terms in the spline could not be interpreted meaningfully, the model was approximately linear in the shortage term and the effect of the shortage could be interpreted as a change in the level of the series. The fitted and observed values of this analysis are shown in Figure 2.

The shortage was estimated to have resulted in a reduction in numbers of heroin possession offences of 74 per month at the time of the shortage, representing a 45% reduction above the effects of the long-term downward trend [7]. The reduction occurred on January 2001, i.e. with a zero month lag. Conclusions were not drawn about the relationship between lags of the two series, since subjectivity in defining the time of onset of the shortage makes this uncertain.

These analyses confirmed the proposition that heroin users switched to cocaine when heroin suddenly became unavailable. This switch was short-lived, however, either because of the greater difficulty in maintaining cocaine rather than heroin use or because cocaine also became less readily available.

## Discussion

Standard ITSA can be extended to understand unexpected and unplanned events, even when the occurrence of such events is only recognized after the fact. By using the case of an abrupt and sustained reduction in the availability of heroin in Australia in January 2001, we have shown that problems associated with applying standard ITSA can be overcome by: defining and dating such an event in ways that are not influenced by the outcomes to be modeled; identifying appropriate indicator series to model; fitting ITSA models to the data in ways that minimize the effects of researcher bias by using a prescriptive method to select functional depictions of the event and subsequently modifying these using appropriate transfer functions; and evaluating plausible rival explanations of any changes observed in the time series so that these changes could be more confidently attributed to the unplanned event.

## Conclusion

This method is particularly useful for unplanned and poorly understood events (such as the heroin shortage) which are of a size and nature that would be difficult or impossible to mimic experimentally, but whose consequences are of potentially great public health interest to researchers and policy makers. Although resource-intensive and detailed, the method presented here provides an opportunity to gain valuable insights. When applied to the heroin shortage, the method has enabled Australian researchers to test theories about the interaction of cocaine and heroin markets [22,23], and to extend current understanding of drug markets to investigate the health and social consequences of sharp changes in the availability of illicit opioids in an environment of effective and widespread harm reduction measures [4,24]. Such findings are of great value to law enforcement, drug treatment and health policy makers, and would have been very difficult to obtain without the aid of the natural experiment provided by the heroin shortage.

## Abbreviations

AIC Akaike Information Criterion

ARIMA Auto-regressive Integrated Moving Average

IDRS Illicit Drug Reporting System

IDU Injecting Drug Users

ITSA Intervention time series analysis

KI Key Informant

## Competing interests

The author(s) declare that they have no competing interests.

## Authors' contributions

All authors 1) have made substantial contributions to conception and design; 2) have been involved in drafting the manuscript or revising it critically for important intellectual content; and 3) have given final approval of the version to be published.

## Pre-publication history

The pre-publication history for this paper can be accessed here:


